# The prevalence of HIV among MSM in China: a large-scale systematic analysis

**DOI:** 10.1186/s12879-019-4559-1

**Published:** 2019-11-27

**Authors:** Meng-Jie Dong, Bin Peng, Zhen-Feng Liu, Qian-ni Ye, Hao Liu, Xi-Li Lu, Bo Zhang, Jia-Jia Chen

**Affiliations:** 1 The Department of Nuclear Medicine, The First Affiliated Hospital, College of Medicine, Zhejiang University, Zhejiang, 310003 People’s Republic of China; 2 Department of Medical Statistics, Chongqing Medical University, Chongqing, 400016 People’s Republic of China; 3 Infectious Disease Department, College of Medicine, Zhejiang University, Zhejiang, 310003 People’s Republic of China

**Keywords:** Prevalence, MSM, HIV, Systematic analysis, China

## Abstract

**Background:**

The prevalence of HIV among men who have sex with men (MSM) has become a significant public health challenge. The aim was to comprehensively estimate the national prevalence of HIV among MSM and its time trends through a large-scale systematic analysis.

**Methods:**

Systematic search of Cochrane Library, PubMed, EMBASE, CNKI, VIP, and Wanfang Data databases without language restriction for studies on the prevalence of HIV among MSM published before Dec.31, 2018. Studies were eligible for inclusion if they were published in the peer-reviewed literature and used validated assessment methods to assess the prevalence of HIV among MSM. Estimates were pooled using random-effects analysis.

**Results:**

Data were extracted from 355 cross-sectional studies (571,328 individuals) covered 59 cities from 30 provinces and municipalities of China. The overall national prevalence of HIV among MSM from 2001 to 2018 was estimated to be 5.7% (95% CI: 5.4–6.1%), with high between-study heterogeneity (*I*^2^ = 98.0%, *P* <  0.001). Our study showed an increased tendency in the HIV prevalence as time progressed by meta-regression analysis (*I*^2^ = 95.9%, *P* <  0.0001). HIV prevalence was the highest in those aged 50 years and older with HIV prevalence of 19.3% (95%CI: 13.1-27.4%, *N* = 13). HIV was more prevalent in the illiterate population (16.8%), than in those who had received an education. Although the internet was a major venue for Chinese MSM seeking male sex partners (35.6, 95%CI: 32.3-39.9%, *N* = 101), seeking MSM in bathhouses/saunas had the highest associated prevalence of HIV (13.4, 95%CI: 10.3-17.1%, *N* = 22). The HIV prevalence among MSM varied by location: compared with other regions in China, HIV was highly prevalent among MSM in the southwest (10.7, 95%CI: 9.3-12.2%, *N* = 91). Compared to participants who sometimes or always used condoms, participants who had never used a condom in the past 6 months had a higher risk of HIV infection, with odds ratios of 0.1 (95%CI: 0.08-0.14).

**Conclusions:**

Our analysis provided reliable estimates of China’s HIV burden among MSM, which appears to present an increasing national public health challenge. Effective government responses are needed to address this challenge and include the implementation of HIV prevention.

## Background

Since the late 1990s, increasing numbers of men who have sex with men (MSM) have been diagnosed with HIV in the majority of countries with large and visible MSM communities [1–3]. Chinese scientists began studying HIV-related risk behaviours among MSM in 1993, but epidemiological studies assessing the prevalence of HIV were not conducted until 2000 [2]. A growing body of evidence from different time periods and locations has shown that MSM play an increasingly important role in China’s HIV/AIDS epidemic. A report by the Chinese Ministry of Health estimated that approximately 780,000 people were estimated to be living with HIV/AIDS in China in 2011, and 17.4% of the estimated HIV/AIDS cases were attributable to male-to-male sexual contact [3]. Recent national reports showed that HIV transmission among MSM accounted for 21.4 to 23.4% of the newly identified HIV/AIDS cases in some areas in China [2, 4, 5].

Estimates of the overall HIV prevalence among MSM in China have relied on several different reviews which conducted from 2001 to 2009 [6], 2003 to 2009 [7], and 2005 to 2010 [8]; these reviews found that MSM formed a high-risk population for HIV infection in China, with an overall prevalence ranging from 2.5 to 6.5%. Furthermore, these reviews identified a rising trend in the national HIV prevalence, with an estimated 0.9% increase in HIV infection among MSM in China from 2003 to 2008 [3, 6–9]. However, our understanding of the nationwide HIV epidemiology remains incomplete. A wide range of demographic, behavioural, and societal factors that predict HIV acquisition among MSM in China have been identified. However, to the best of our knowledge, the relationships between these factors (including age, education, sex partners, commercial sex, and unprotected sexual intercourse) and the prevalence of HIV infection among MSM have not been reported based on all available data from China.

An accurate understanding of the HIV prevalence among MSM is critical for tailoring interventions and evaluating previously established programmes and services. The purpose of this study was to perform a large-scale systematic analysis to examine HIV epidemiology among MSM in China based on studies published up to Dec.2018.

## Methods

### Search strategy

A comprehensive literature search was conducted in the following databases to identify studies published up to Dec.31, 2018: Cochrane Library, PubMed, EMBASE, Chinese National Knowledge Infrastructure (CNKI), VIP, and Wanfang Data. Two independent investigators performed the searches in parallel. We used combinations of keywords and medical subject heading (MESH) terms as follows: (“HIV” or “AIDS” or “human immunodeficiency virus” or “acquired immunodeficiency syndrome”) and (“homosexual” or “gay” or “men who have sex with men” or “MSM” or “bisexual” or “Tongzhi” [the Chinese term referring to homosexual men]) and (“China” or “Chinese”) and (“prevalence” or “infection” or “associated risk” or “infection status” or “epidemic status” or “surveillance”). We also searched relevant reference lists and relevant journals manually, which were not mentioned in the introduction, and corresponded with authors to obtain the original data if necessary. We did not include grey literature (i.e., literature that had not been formally published). This systematic analysis adhered to the Preferred Reporting Items for Systematic Reviews and Meta-Analyses (PRISMA) guidelines [10].

### Inclusion and exclusion criteria

Studies with the following criteria were included to assess the prevalence of HIV among MSM: (i) studies involving MSM in mainland China; (ii) studies that required both presumptive and confirmatory tests for the HIV diagnosis; (iii) the English version of a study if the study was reported in both Chinese and English; and (iv) the comprehensive article (i.e., prevalence of HIV and its possible related risk factors) if multiple publications reported results based on the same research. Data from eligible studies were extracted by two reviewers independently.

We excluded duplicate studies within and between databases, studies with no numerical estimates, studies of Chinese populations conducted outside of mainland China, reviews, and viewpoints. We excluded animal studies and studies with a self-reported HIV status unconfirmed by tests. Studies with a sample size less than 30 were excluded [7]. Disagreements were resolved by discussion. If no consensus could be reached, the issue was referred to a third author.

### Quality assessment

The quality assessment tool for systematic reviews of observational studies (QATSO) was selected to evaluate the quality of the included studies, and a validated quality assessment tool was used to assess HIV prevalence/risk behaviours among MSM [11]. Items were scored as 0, 1, and NA, representing “no,” “yes,” and “not applicable,” respectively. The total score of each eligible study had to be above 33% (scores of 0 to 33%, 33 to 66%, and 67 to 100% represented “poor” “satisfactory,” and “good” quality studies, respectively.)

### Statistical analysis

We calculated the prevalence estimates with logit transformation, and a systematic analysis was conducted to calculate the pooled estimates following the methods suggested by DerSimonian and Laird [12, 13]. Clopper-Pearson confidence intervals were calculated for all prevalence estimates. An analysis of heterogeneity was performed using Cochran’s Q test (*p* <  0.10 indicating significant heterogeneity) and the *I*^*2*^ statistic. *I*^*2*^ values of 25, 50, and 75% represented low, moderate, and high degrees of heterogeneity, respectively. If the data were heterogeneous, random-effects models were used for the systematic analysis [14].

Many factors can affect homogeneity between studies. Therefore, we performed subgroup analyses to explore the potential sources of between-study heterogeneity. We calculated the prevalence of HIV among MSM by age, education, marital status, geographical distribution, occupation, gender of first sexual partner, and condom use in the last 6 months. Potential publication bias was assessed for significance using Begg’s test and was graphically explored by generating funnel plots.

All analyses were performed using R version 3.2.2 [15, 16].

## Results

**Fig. 1:**
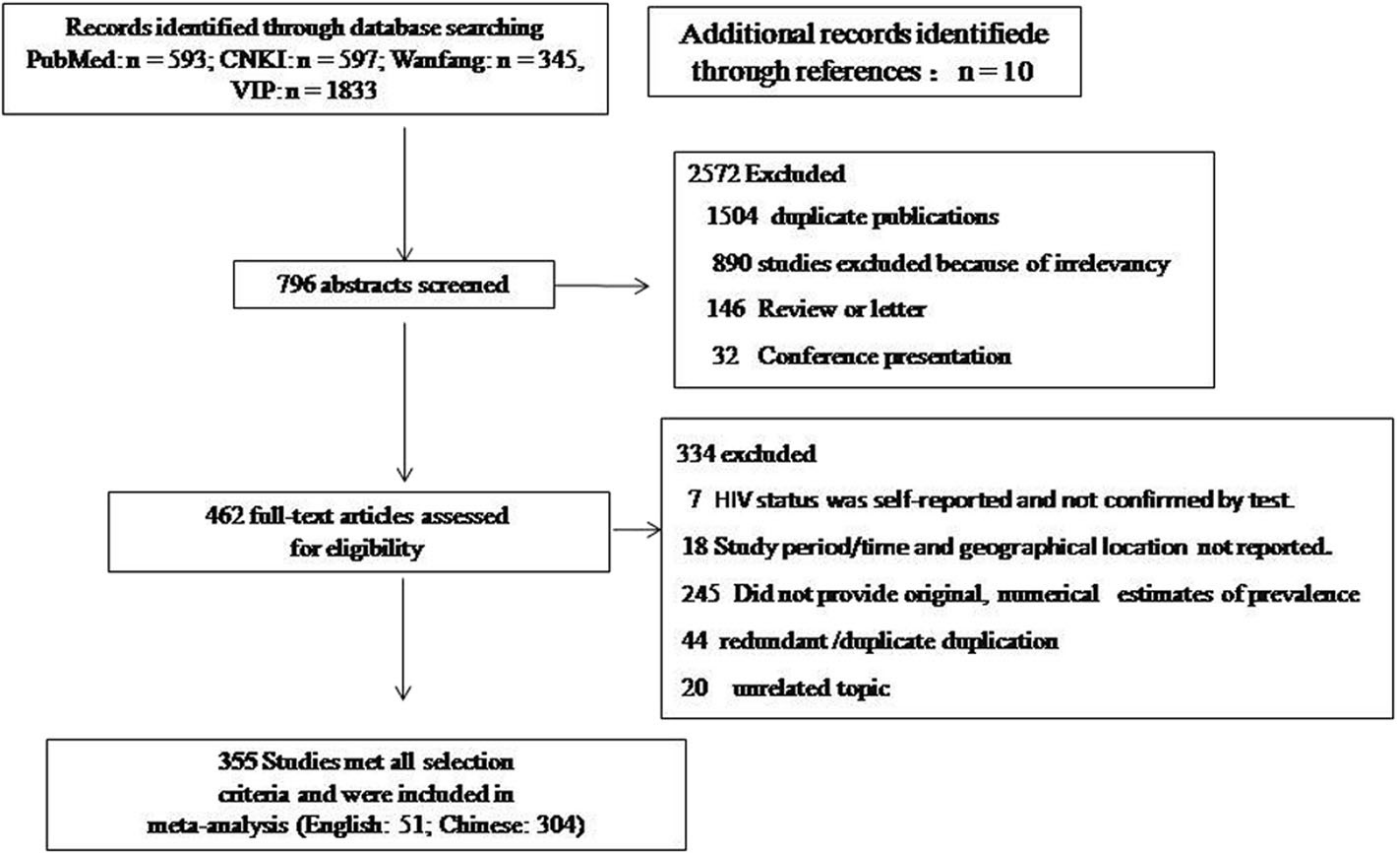
Flow chart showing the selection process for studies included in the systematic analyses

As shown in Fig. 1, a total of 3368 relevant articles were identified, of which 760 were further screened, and 355 cross-sectional studies (51 published in English and 304 in Chinese, which included 571,328 MSM) were finally included in the systematic analysis [10, 11, 14–366]. The characteristics of the included studies are summarized in Table 1.

**Table 1:** Characteristics of Study Participants (355 studies)

Variable	Number of studies	n or n/N	Percentage
Age (years_._)			
<20	93	8856/102,298	4.6% [3.8%; 7.3%]
20-29	86	43.354/80,249	55.3% [52.9%; 57.6%]
30-39	85	19,141/73,768	24.7% [23.5%; 25.9%]
40-49	41	3643/34,063	10.2% [9.0%; 11.4%]
50+	39	1584/42,046	3.2% [2.6%; 4.1%]
Sample size			
<100	15	1130	0.2%
101-200	41	6372	1.1%
201-500	138	49,243	0.8%
501-1000	63	45,714	8.0%
1001-2000	58	83,815	14.7%
2001-5000	25	76,743	13.4%
5001-10,000	9	63,424	11.1%
>10,001	6	244,887	42.9%
Marital status			
Single	249	183,832/267,519	68.7% [65.3%; 73.6%]
Cohabitating	98	3508/129,646	2.7% [2.1%; 3.1%]
Married	193	153,060/335,726	22.3% [19.8%; 25.0%]
Divorced or widowed	195	11,126/224,119	5.0% [4.2%; 5.3%]
Current level of education			
Illiterate	38	316/34,779	0.8% [0.6%; 1.3%]
Primary school	74	1371/42,411	3.21% [2.7%; 3.8%]
Junior high school	117	17,979/95,030	19.1% [17.8%; 20.5%]
Senior high school	212	84,723/245,166	34.4% [33.5%; 35.5%]
College or above	250	180,806/418,465	43.2% [42.7%; 45.2%]
Sexual orientation			
Homosexual	143	53,653/92,721	58.9% [57.3%; 60.4%]
Bisexual	116	23,117/65,545	33.8% [32.1%; 35.6%]
Heterosexual	85	2370/47,974	2.8% [2.1%; 3.7%]
Undetermined	71	2063/38,423	5.7% [4.9%; 6.7%]
Main location to seek homosexual partners			
Internet	101	112,979/316,944	35.6% [32.3%; 39.9%]
Parks	85	17,547/141,598	12.4% [10.9%;14.3%]
Public bathhouses/saunas	98	40,029/303,129	13.2% [12.4%; 14.5%]
Bar/night club/tearoom	100	42,978/165,416	25.9% [22.4%; 27.0%]
Sampling methods			
Respondent driven sampling (RDS)	39	28,952	5.0%
Snowball	123	271,041	47.0%
Time-venue	82	95,268	16.3%
Convenience	11	6828	1.2%
Multiple methods	39	121,732	20.2%
Other or not defined	61	58,895	10.3%
Condom use (in the last 6 months)			
During sex with men			
Always	163	113,092/280,617	41.5% [40.1%; 42.8%]
Sometimes	119	46,927/90,253	48.3% [46.7%; 50.0%]
Never	101	7890/86,014	10.1% [9.0%; 11.4%]
During commercial sex with men			
Always	63	8622/19,537	52.1% [48.2%; 56.0%]
Sometimes	33	1274/3549	33.6% [29.5%; 38.0%]
Never	33	294/3533	9.0% [6.0%; 13.4%]
When purchasing sex			
Always	9	282/623	50.4% [39.3%; 61.6%]
Sometimes	5	190/487	34.0% [25.7%; 43.3%]
Never	5	69/487	14.3% [10.1%; 19.7%]
During anal sex with men when selling sex			
Always	10	538/913	60.7% [52.2%; 68.7%]
Sometimes	5	234/669	31.2% [22.8%; 40.9%]
Never	5	62/669	10.7% [6.3%; 17.5%]
During sex with a woman			
Always	108	16,282/61,944	29.6% [28.1%; 31.1%]
Sometimes	68	5396/16,474	34.3% [31.4%; 37.3%]
Never	68	5937/17,665	34.2% [31.2%; 37.4%]
Occupation			
Student	81	8592/56,128	16.3% [14.3%; 18.5%]
Teacher	20	150/5460	3.0% [2.4%; 3.8%]
Office staff	51	8946/39,117	13.9% [11.4%; 16.7%]
Farmer	22	833/19,603	4.2% [2.4%; 7.4%]
Service business employee	63	14,358/51,468	25.7% [22.8%;28.9%]
Jobless or job-seeking	26	1558/15,119	9.2% [6.7%; 12.5%]
Worker	46	4793/36,243	13.0% [11.3%; 14.9%]
Retired	7	41/2591	1.9% [0.8%; 4.3%]
Food and beverage service personnel	20	573/8375	6.0% [4.3%; 8.5%]
Sexual debut partner			
Male	54	23,608/38,680	60.2% [57.8%; 62.6%]
Female	54	14,547/38,680	38.0% [35.5%; 40.5%]
HIV/AIDS-related knowledge			
Awareness rate of HIV/AIDS knowledge	104	225,951/254,330	91.1% [89.5%; 92.4%]
HIV can be transmitted through the blood or blood products	70	29,812/31,420	95.7% [94.6%; 96.5%]
HIV can be sexually transmitted	73	28,601/30,682	97.6% [95.7%; 98.7%]
HIV can spread through mosquito or other insect bites	62	20,718/28,888	74.2% [70.7%; 77.4%]
People who look healthy can still have HIV	61	20,801/27,084	82.2% [78.5%; 85.4%]
A pregnant woman with HIV can transmit the virus to her baby	70	26,869/29,382	92.8% [91.0%; 94.3%]
Sharing needles for drug use with someone who has	58	24,262/25,621	95.7% [94.5%; 96.6%]
HIV or AIDS could cause HIV infection	63	24,072/29,093	87.1% [84.3%; 89.4%]
**Drug use**			
Drug use	83	1044/59,764	1.5% [1.1%; 2.0%]
No drug use	20	16,783/17,268	97.6% [95.7%; 98.7%]

Figures in parentheses are 95% CIs; n, number of MSM in the subgroup; N, total number investigated in the subgroup_._

### Characteristics of the selected studies

The 355 eligible articles included in this study covered 59 cities from 30 provinces and municipalities of China (no studies were from Tibet). The sample size of the selected studies ranged from 30 to 1,498,841 (mean 4373, 95% confidence interval [CI]: 455 – 8290) [24, 25]. Among the 355 selected articles, 6 reported results for multiple study sites, and 79 reported HIV prevalence estimates for more than one time period, resulting in a total of 581 HIV prevalence estimates. The first included study was reported in 2001 [19], with an HIV prevalence of 1.31%, and the studies provided overall estimates of the HIV prevalence among MSM in China from 2001 to 2018.

The sampling methods varied, with 123 studies using snowball sampling, 39 studies using respondent-driven sampling (RDS), 82 studies sampling from time-venue, 39 studies using multiple recruitment methods, and 11 applying convenience sampling methods. The analysed data indicated that the largest proportion (35.6, 95% CI: 32.3-39.9%) searched for sexual partners on the internet; among the other locations, 25.9% (95% CI: 22.4-27.0%) searched at bars/night clubs/tearooms, 13.2% (95% CI: 12.4-14.5%) searched at public bathhouses/saunas, and 12.4% (95% CI: 10.9-14.3%) searched at parks.

**Fig. 2:**
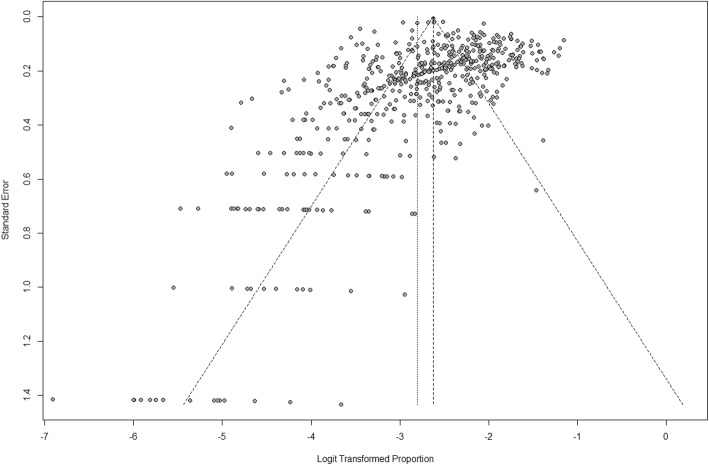
Funnel plot showing the potential publication bias

We found a publication bias, the problem that results from systematic differences between the results of all the completed studies on a topic and the results of the subset of those studies that are published, across the studies reporting the HIV prevalence (*t* = − 4.12, *p* = 0.0011) (Fig. 2)*,* which must be taken especially seriously, as it presents perhaps the greatest threat to the validity of this method.

### Study quality assessment

In the quality assessment, 214 of the included studies were considered “good” quality (values between 67 and 100%), 141 were considered “satisfactory” (values between 33 and 66%), and none were considered “poor” (values between 0 and 33%).

### Demographic information

The demographic characteristics are detailed in Table 1. Most participants (68.7%) were single, although 22.3% had married a woman, 2.5% were cohabiting with their same-sex partner, and 5.0% were divorced or widowed. The sexual behaviour data indicated that 58.9% exclusively had sex with men, 33.8% had sex with both men and women, 2.8% were heterosexual, and 5.7% were undetermined.

### Prevalence of HIV infections among MSM

Overall, the national HIV prevalence among MSM from 2001 to 2018 was 5.7% (95% CI: 5.4–6.1%, *N* = 355), with study prevalence rates ranging from 0% (95% CI: 0.1-2.5%) to 22.91% (95% CI: 18.1-28.3%) [17, 341]. Substantial heterogeneity existed between the studies (*p* for Q test, *p <* 0.0001; *I*^2^ = 98.0%).

### Chronological prevalence of HIV infections among MSM

**Fig. 3:**
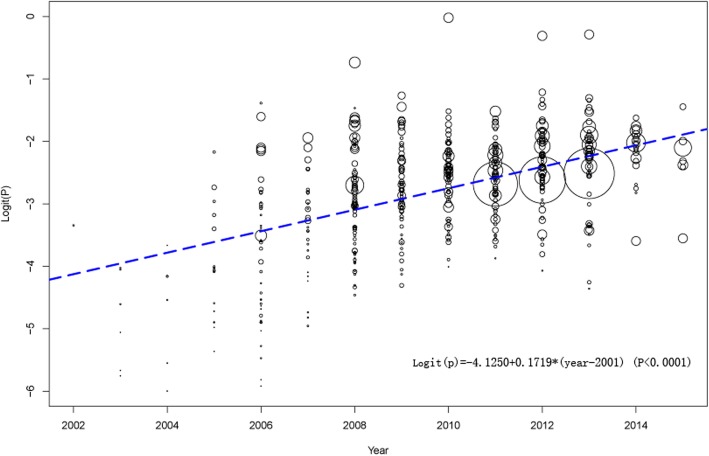
Chronological prevalence of HIV infection among MSM analysed by meta-regression

Based on the study years selected for our investigation, we estimated chronological prevalence of HIV in China by meta-regression analysis (*I*^2^ = 95.9%, *P* <  0.001), demonstrating an increased tendency as time progressed (Fig. 3).

### Relationships between HIV prevalence and age, education, marital status, occupation, ethnicity, and sexual debut partner

Table 2 shows the age-specific prevalence of HIV. For each 10-year increase in age, the prevalence of HIV also increased from 4.6% in people aged < 20 years to 7.2% in people aged 20-29 years to the highest prevalence (19.3%) in those aged > 50 years.

**Table 2:** Stratified analyses of HIV prevalence among MSM in China

Variable	Number of studies	HIV prevalence	Heterogeneity of included studies			
			***I***^***2***^	Q	***p***	OR
Age (yrs.)						
<20	33	4.6% [3.4%; 6.3%]	64.0%	79.4	< 0.0001	
20-29	32	7.2% [5.9%; 8.7%]	91.6%	276.2	< 0.0001	
30-39	30	11.8%[10.0%; 13.8%]	75.2%	120.8	< 0.0001	
40-49	30	13.8%[11.0%; 17.1%]	51.8%	37.4	0.0047	
50+	13	19.3%[13.1%; 27.4%]	50.2%	26.1	0.0166	
Sampling methods						
RDS	39	5.4% [4.7%; 6.3%]	90.3%	627.4	< 0.0001	
Snowball	126	6.4% [5.9%; 7.0%]	96.6%	3132.0	< 0.0001	
Time-venue	83	5.4% [4.6%; 6.3%]	96.9%	2694.4	< 0.0001	
Convenience	11	4.2% [3.3%; 7.3%]	96.2%	173.5	< 0.0001	
Multiple methods	42	5.0% [3.9%; 6.4%]	99.0%	3762.7	< 0.0001	
Marital status						
Single	57	6.4% [5.5%; 7.5%]	94.5%	876.3	< 0.0001	
Cohabitating	21	11.5% [7.8%; 16.5%]	67.8%	65.3	< 0.0001	
Married	42	10.6% [9.2%; 12.0%]	72.5%	152.9	< 0.0001	
Divorced or widowed	49	12.8%[10.0%; 16.4%]	81.6%	240.9	< 0.0001	
Current level of education						
Illiterate	3	16.8% [6.4%;37.3%]	0.0%	0.8	0.6683	
Primary school	11	15.6% [11.0%;21.6%]	25.4%	14.7	0.1948	
Junior high school	20	9.8% [7.4%; 12.9%]	86.8%	151.8	< 0.0001	
Senior high school	50	7.6% [6.4%; 9.0%]	89.5%	428.6	< 0.0001	
College or above	62	5.7% [5.0%; 6.5%]]	87.7%	464.9	< 0.0001	
Sexual orientation						
Homosexual	23	7.0% [5.4%; 9.1%]	93.4%	540.3	< 0.0001	
Bisexual	10	6.1% [4.5%; 8.3%]	69.0%	32.2	0.0004	
Heterosexual	11	7.5% [4.4%; 12.7%]	59.3%	27.0	0.0045	
Undetermined	7	7.6% [3.2%; 16.8%]	0.0%	15.4	0.0175	
Main location to seek homosexual partners						
Internet	20	7.3% [5.8%; 9.1%]	94.2%	314.7	< 0.0001	
Parks	19	6.0% [4.8%; 7.4%]	38.2%	38.2	0.0037	
Public bathhouses/saunas	22	13.4%[10.3%; 17.1%]	88.6%	162.3	< 0.0001	
Bar/night/club/tearoom	23	7.4% [5.8%; 9.3%]	98.9%	141.2	< 0.0001	
Ethnicity						
Han Chinese	15	6.3% [4.9%; 8.0%]	95.6%	317.7	< 0.0001	0.78[0.30,2.03]
Other	15	7.0% [3.4%; 14.0%]	93.6%	218.9	< 0.0001	
Condom use (in the last 6 months)						
During sex with men						
Always	27	4.4% [3.3%; 5.8%]	91.1%	321.5	< 0.0001	
Sometimes	27	8.2% [6.3%; 10.5%]	96.0%	510.5	< 0.0001	
Never	27	9.9% [7.4%;13.1%]	84.5%	145.1	< 0.0001	
During commercial sex with men						
Always	15	2.9% [1.6%; 5.0%]	69.8%	39.8	< 0.0001	
Sometimes	15	6.6% [4.0%; 10.7%]	85.2%	59.8	< 0.0001	
Never	14	8.5% [5.3%; 13.2%]	26.3%	23.7	0.0345	
Condom use during sex with a woman in the past 6 months						
Always	11	4.1% [2.5%; 6.4%]	48.1%	20.5	0.021	
Sometimes	9	3.4% [1.7%; 6.6%]	80.2%	42.4	< 0.0001	
Never	10	5.9% [3.2%; 10.7%]	86.9%	71.4	< 0.0001	
Occupation						
Student	12	6.1% [4.5%; 8.4%]	59.8%	29.8	0.0029	
Teacher	2	5.3% [1.1%; 22.8%]	0.0%	0.6	0.7348	
Office staff	12	8.0% [6.2%; 10.2%]	76.2%	50.5	< 0.0001	
Farmer	2	14.8% [3.8%; 43.2%]	93.4%	30.3	< 0.0001	
Service business employee	8	8.1% [5.6%; 11.6%]	90.3%	82.7	< 0.0001	
Jobless or job-seeking	9	10.3% [8.4%; 12.6%]	31.2%	13.1	0.159	
Worker	8	8.6% [7.5%; 9.9%]	33.1%	12.0	0.1533	
Sexual debut partner						
Male	8	6.8% [4.0%; 10.1%]	95.4%	152.8	< 0.0001	0.60[0.53,0.69]
Female	8	10.2% [6.6%; 14.4%]	94.5%	127.4	< 0.0001	
Drug use						
Yes	7	6.4% [4.2%; 10.0%]	42.9%	10.5	0.1046	1.14[0.31,4.21]
No	7	8.2% [2.4%; 17.0%]	99.2%	770.7	< 0.0001	
Quality ssessment						
satisfactory	142	5.5% [4.6%; 6.6%]	96.5%	2481.5	< 0.0001	0.74[0.60, 0.92]
good	213	7.4% [6.5%;8.4%]	97.7%%	1823.0	< 0.0001	

Figures in parentheses are 95% CIs; OR, odds ratio

The prevalence of HIV decreased with increasing years of education, with a prevalence in the illiterate group of 16.8% (95% CI: 6.4-37.3%), which was higher than the prevalence rate among those who had received an education. Of the assessed occupations (including teacher, office staff, farmer, service business employee, unemployed and job-seeking, and worker), the prevalence of HIV was highest among farmers at 14.8% (95% CI: 3.8-43.2%).

The prevalence rates of HIV by sexual orientation were 7.0, 6.1, 7.5, and 7.6% for the homosexual, bisexual, heterosexual, and undetermined groups, respectively. Although the internet was a major venue for seeking male sex partners among Chinese MSM (35.6, 95% CI: 32.3-39.9%, *N* = 101), MSM seeking male sex partners in bathhouses/saunas had the highest prevalence of HIV (13.4, 95% CI: 10.3-17.1%, *N* = 22).

The odds ratio (OR) of HIV for those whose first sexual encounter was with a male compared to those with a first sexual encounter with a female was 0.6 (95% CI: 0.5-0.7), suggesting that in China, MSM with a female sexual debut partner had a higher HIV prevalence than those with a male sexual debut partner. Drug use was not a significant contributor to HIV transmission among Chinese MSM (OR: 1.14, 95% CI: 0.31-4.21).

### Geographical characteristics of the HIV prevalence

**Fig. 4:**
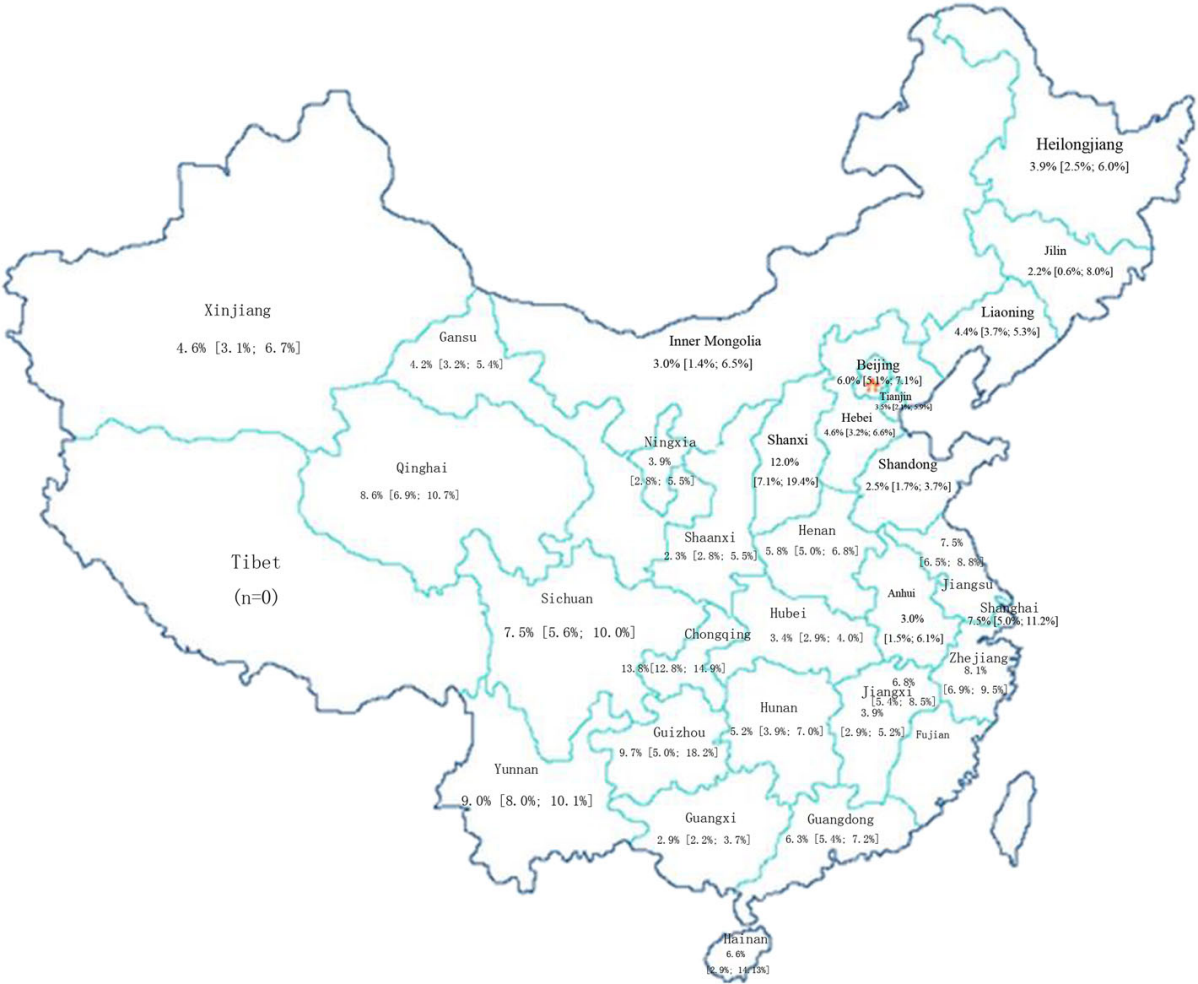
Map of the geographical characteristics of the HIV prevalence across China

To determine the geographical characteristics of the HIV prevalence in China, we analysed the differences in prevalence by geographical divisions in China based on its provinces or municipalities. The number of studies, total HIV-positive population, and pooled sample size were summarized for the different geographical divisions (Fig. 4).

Overall, the highest pooled HIV prevalence among MSM was found in southwest China (10.7, 95% CI: 9.3-12.2%, *N* = 91), in which Chongqing city had the highest HIV prevalence (13.8, 95% CI: 12.8-14.9%, *N* = 24), followed by east China (6.3, 95% CI: 5.6-7.0%, *N* = 167), which had a prevalence higher than the national average. The pooled HIV prevalence among MSM in northeast China (4.2, 95% CI: 3.4-5.0%, *N* = 58), north China (5.2, 95% CI: 4.4-6.1%, *N* = 66), northwest China (3.7, 95% CI: 3.0-4.5%, *N* = 57), and south China (5.1, 95% CI: 4.4-5.9%, *N* = 121) were lower than the prevalence of the country as a whole.

### Condom use information (in the last 6 months)

Participants who had engaged in unprotected sex in the past 6 months had a higher risk of HIV infection than those who reported protected sex (either sometimes or always using a condom). The ORs for participants who never used a condom during sex with men, during commercial sex with men, and during sex with a woman in the past 6 months were 0.11 (95% CI: 0.08-0.14), 0.11 (95% CI: 0.09-0.13), and 0.07 (95% CI: 0.03-0.14), respectively.

## Discussion

### Study findings

To the best of our knowledge, this study is the first large-scale systematic analysis of the epidemiology of HIV infection among MSM in China up to 2018. The study included 355 eligible studies that covered 59 cities from 30 provinces and municipalities. All the studies were of “satisfactory” or “good” quality, and the results of these studies were heterogeneous. Significant publication bias (Begg’s test, *t* = − 4.12, *p*-value = 0.0011) existed across the studies reporting the prevalence of HIV. The national estimate of HIV prevalence among MSM from 2001 to 2018 was 5.7% (95% CI: 5.4–6.1%), with study prevalence rates ranging from 0% (95% CI: 0.1-2.5%) to 22.9% (95% CI: 18.1-28.3%) [17, 338]. The prevalence of HIV among MSM increased substantially between 2001 and 2018. These findings might have important policy implications because the prevalence also differed by age, education, occupation, and condom use. The data from this study indicated that the HIV prevalence differed by region, and a high burden of HIV infection was observed among MSM in southwest China, especially in Chongqing city. Although the internet was a major venue used by Chinese MSM to search for male sex partners, MSM who sought partners at bathhouses, saunas, and massage rooms had the highest HIV prevalence. Studies using snowball sampling had a higher prevalence of HIV infection than studies using RDS, convenience sampling, or multiple sampling methods. A possible explanation for this finding is that individuals with high-risk behaviours are more likely to accept HIV testing through snowballing sampling [2].

The present study provides insights into the national HIV prevalence among MSM over the past 18 years and proposes a suggestion for China’s health department to implement more effective prevention strategies and policies in the future. The national estimate of HIV prevalence in China differed somewhat from those of other countries or regions. For example, the prevalence of HIV among MSM is estimated to be 19% in the United States [367], 14.2% in Brazil, 3.0% (95% CI: 2.4–3.6) in the Middle East and North African region, 6.56% (95% CI: 5.54-7.57) in eastern Europe and central Asia, 14.74% (95% CI: 14.05-15.42) in south and southeast Asia, and 25.4% in the Caribbean [368]. Our study showed an increased tendency in the HIV prevalence as time progressed (*P* <  0.001) (Fig. 2). The increasing HIV prevalence may be associated with several factors: 1) the increasing migration for better employment opportunities and living conditions from regions with a high HIV prevalence, such as from southern China or from the countryside, to large cities, which have a relatively open culture and convenient sexual venues (e.g., bars, saunas, parks, and sex clubs.) [203]; 2) the gradual changes in attitudes towards sex and increased openness of male homosexuality in China with changes in Chinese society, although homosexuality is still not widely accepted by the general population [369]; and 3) the common occurrence of marriage between MSM and women because MSM might act as a bridge for HIV transmission from other MSM to the general population [9]. Additionally, to some extent, the increased HIV prevalence may be due to an increase in the testing rates because HIV testing among Chinese MSM has increased over the past decade.

### HIV prevalence in terms of age

A better understanding of the mechanisms underlying the age-related risks of HIV infection can help address the situation in practice. The findings presented in this article confirmed the notable increase in the HIV prevalence with increasing age in China because HIV was most prevalent in those aged 50 years and older; these results demonstrated a prominent age-dependent increase in HIV. The distribution of HIV cases by age differed somewhat from that of other countries [370]. A higher HIV prevalence was found among MSM aged 15–19 and 20–24 years in the United States and among 15- to 24-year-old MSM in the United Kingdom [369, 371]. The reason for the increasing HIV prevalence in older MSM may be that older MSM have longer durations of exposure to HIV; additionally, unprotected anal intercourse (UAI) is more common among older MSM than in their younger counterparts, who may also have received a higher level of education [372]. Therefore, behavioural scientists and practitioners alike must address the implications of these findings when developing targeted prevention interventions and treatment services for older MSM [373].

Importantly, the largest subgroup of MSM in this study was those aged 20-29 years (55.3% of the MSM, 95% CI: 52.9-57.6%), which is similar to findings in the literatures [1, 112, 370]. Although younger MSM may be over sampled, the phenomenon should be taken seriously because younger MSM are sexually active, less able than older MSM to negotiate safer sex with their partners and more likely to have multiple sex partners and engage in more complicated sexual networks. Therefore, China may face a widespread HIV epidemic among young MSM if future timely interventions targeting this population are not implemented.

### Relationship between HIV prevalence and geographic location, education, sought sexual partners and occupation

The HIV prevalence among MSM varied according to geographic location. For example, there was an extraordinarily high HIV prevalence of MSM in the southwest compared to the other regions of China (southwest China: 10.7%; northwest China: 3.7%; central China: 5.1%; northeast China: 4.2%; north China: 4.7%; and east China: 6.3%). The high HIV prevalence among MSM in the southwest may be due to several factors: 1) the difference in socioeconomic development between the southwest and other regions of China because the southwest region has a lower economic status and its residents have a lower education level [95]; 2) southwest China includes several areas with a high HIV prevalence, such as Chongqing, which is a city that is accepting towards homosexuality and has very open attitudes about sex [226]; and 3) the increased illicit drug use among MSM in southwest China because drug use is a major risk for HIV transmission among MSM and can contribute to the HIV epidemic [50]. Therefore, to better curb the spread of HIV, targeted measures should be adopted that consider the risk profile of MSM in areas of China with a high HIV prevalence.

Our study found that those who had less education, sought sexual partners at bathhouses or saunas, were farmers and were divorced or widowed were more likely to be infected with HIV. Regarding economic considerations, MSM with less education experience a lack of appropriate health messages and support and are more inclined to seek partners at bathhouses, saunas, or massage rooms due to their low costs [260]. The prevalence of HIV among farmers was 14.8%, which was the highest of all occupations examined. One possible explanation for this high prevalence is that farmers have a lower chance of receiving an education than other occupations in China. Yang et al. found that condom use was clearly higher among MSM with a higher level of education, such as college students or teachers, than that among others and that these more educated groups could volunteer to promote HIV intervention efforts and facilitate a reduction in the HIV infection rate among MSM [374].

### HIV prevalence and sexual risk behaviours

Our study indicated that 22.3% of MSM were married to women; these MSM may conceal their sexual orientation due to the traditional values and social stigma present in China, making it unlikely that they can be reached by traditional prevention measures targeting the general MSM population. Although the proportion of divorced or widowed MSM was only 4.6%, the HIV prevalence appeared to be higher (12.8%) in this population than in the single, married, and cohabitating groups. This higher prevalence may be because MSM who are divorced or widowed no longer have access to a legitimate, routine sexual life and are thus more prone to illegal sexual behaviour and have greater exposure to high-risk environments [327].

Interestingly, HIV knowledge was high in the MSM population (91.1, 95% CI: 89.5-92.4%), but consistent condom use was low, reflecting the complexity of the hypothesis that knowledge transfer and behavioural change are keys to HIV prevention. The study revealed that the rate of consistent condom use was lower when MSM had sex with women (29.6%) than when they had sex with men (41.5% for sex with men and 52.1% for commercial sex with men) over the past 6 months (Table 1). Therefore, MSM are vulnerable to HIV infection from both genders and can serve as a bridge for HIV transmission from one gender to the other. Han et al. reported that MSM who had sex with women were more likely to be married and that once they were HIV carriers, they were likely to exhibit commercial sexual behaviour [119]. This study confirmed that in addition to distribution of accurate and up-to-date information on risky behaviours and effective community-based prevention programmes that make condoms available and accessible, concrete strategies that illustrate and highlight the harm and dangers of not using a condom during penetrative sex need to be implemented to enhance individuals’ motivations to change their behavioural patterns and skills and reduce their HIV risk [375].

Our data indicated that drug use was not a significant contributor to HIV transmission among Chinese MSM (OR: 1.14, 95% CI: 0.31-4.21), suggesting that drug use did not significantly contribute to overall HIV transmission among MSM. However, the increased illicit drug use among Chinese MSM may perpetuate the HIV epidemic, and this relationship may be similar to those in Western countries where drug use is a major risk for HIV transmission [376].

### Limitations

This systematic analysis had several limitations. First, the scarcity of existing research did not allow for subgroup analyses of HIV prevalence by number of sexual partners or for analyses of the differences in condom use among MSM when purchasing or selling sex. Second, this study represented a wide spectrum of MSM in China; the included studies used snowball sampling, RDS, time venue sampling, convenience sampling, and multiple sampling methods. However, few studies in Jilin province (*n* = 2) and Tibet (*n* = 0) were included, which might have affected the regional HIV prevalence among MSM. Finally, significant publication bias (*p*-value = 0.0011) was observed in our analysis. Differences between sampling methods, sample sizes, study locations, and the quality of studies may explain publication heterogeneity. Therefore, readers should be aware that they may be viewing a biased sample of experimental results and should moderate the strength of the conclusions accordingly [377]. Despite the limitations described above, this study employed strict inclusion criteria and applied a valid search strategy to provide an objective, authentic, and current estimate of HIV in China based on a large sample size.

## Conclusions

HIV among MSM is a significant public health challenge in China. Our results showed an increased tendency as time progressed from 2001 to 2018, a higher prevalence of HIV among older MSM than young MSM, and a decreased prevalence with increasing education. These findings illustrate the need for HIV prevention, surveillance, treatment, and intervention strategies among at-risk populations, including evidence-based policy decisions to expand available programmes.

## Data Availability

The datasets used and/or analysed during the current study are available from the corresponding author on reasonable request.

## Abbreviations

AIDS: Acquired immunodeficiency syndrome
CI: Confidence interval
CNKI: Chinese National Knowledge Infrastructure
HIV: Human Immunodeficiency Virus
MSM: Men who have sex with men
